# Supplementary material for: The influence of prior use of inhaled corticosteroids on COVID-19 outcomes: A systematic review and meta-analysis
Source: PLoS One. 2024 Jan 19;19(1):e0295366. doi: 10.1371/journal.pone.0295366 (PMC10798539; doi:10.1371/journal.pone.0295366)

**S1 Fig.** Leave-one-out sensitivity test of the risk of mortality (A), ICU admission (B), hospitalization (C), mechanical ventilation use (D) and length of hospital stay (E) between inhaled corticosteroid (ICS) use and non-use.

(A)


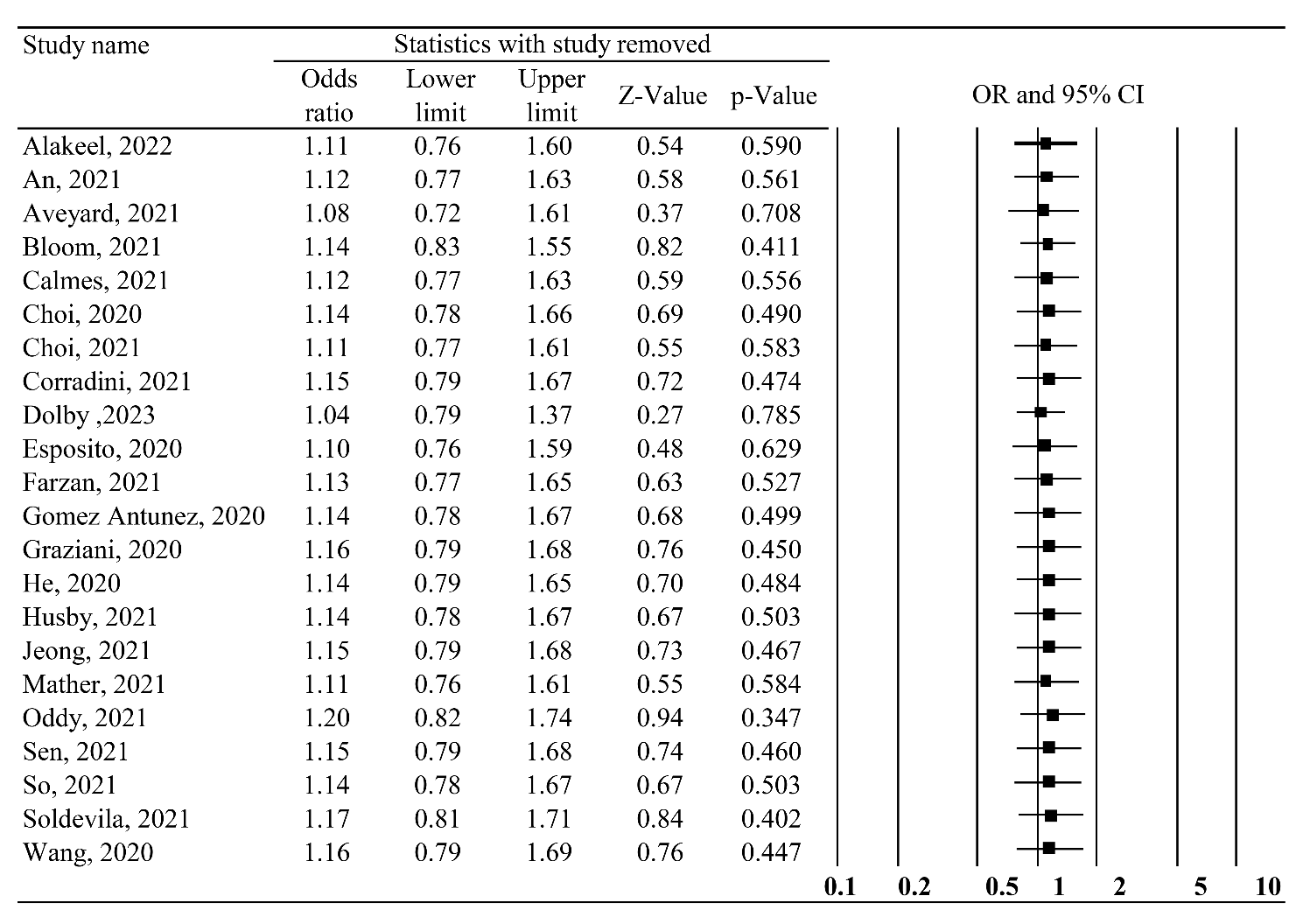


(B)


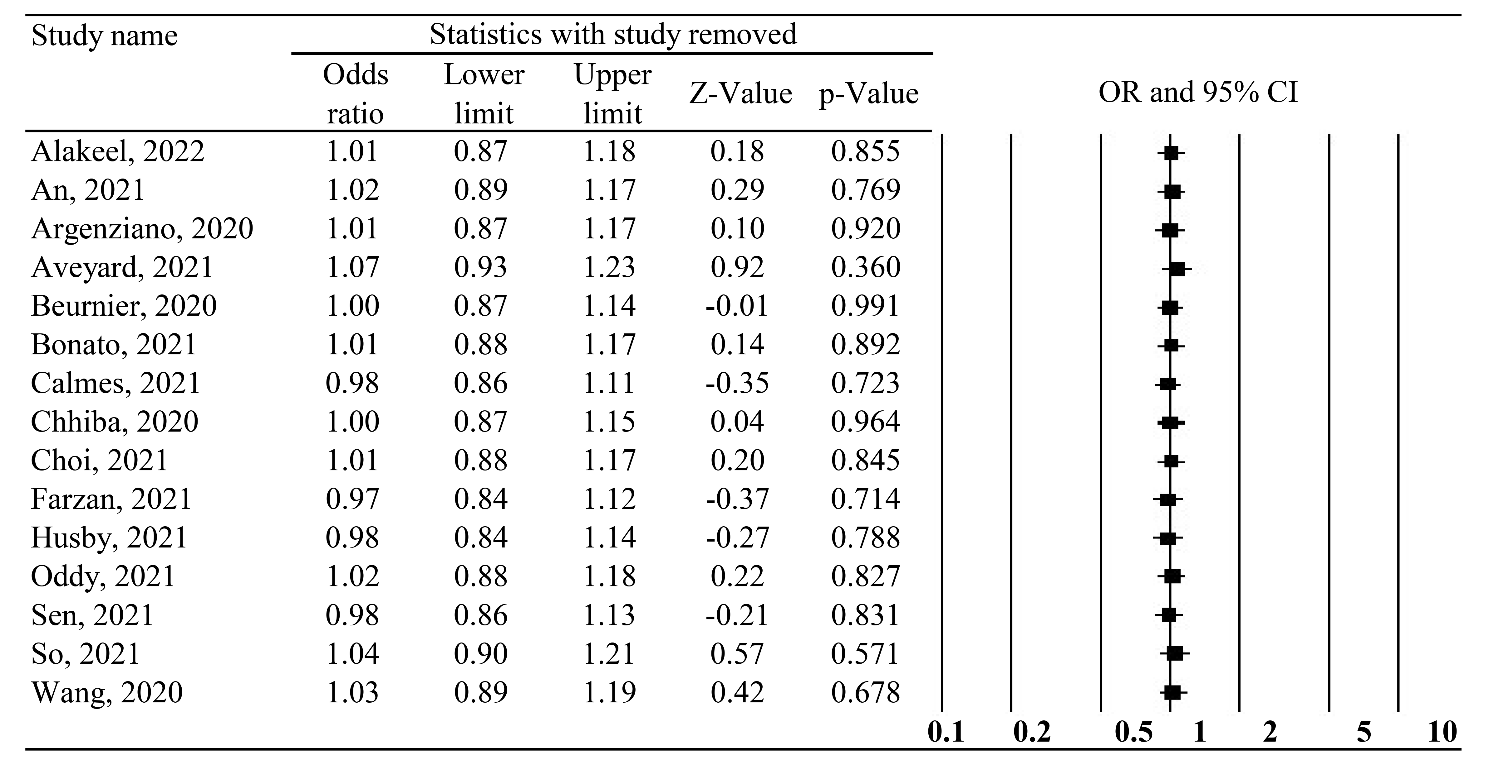


(C)


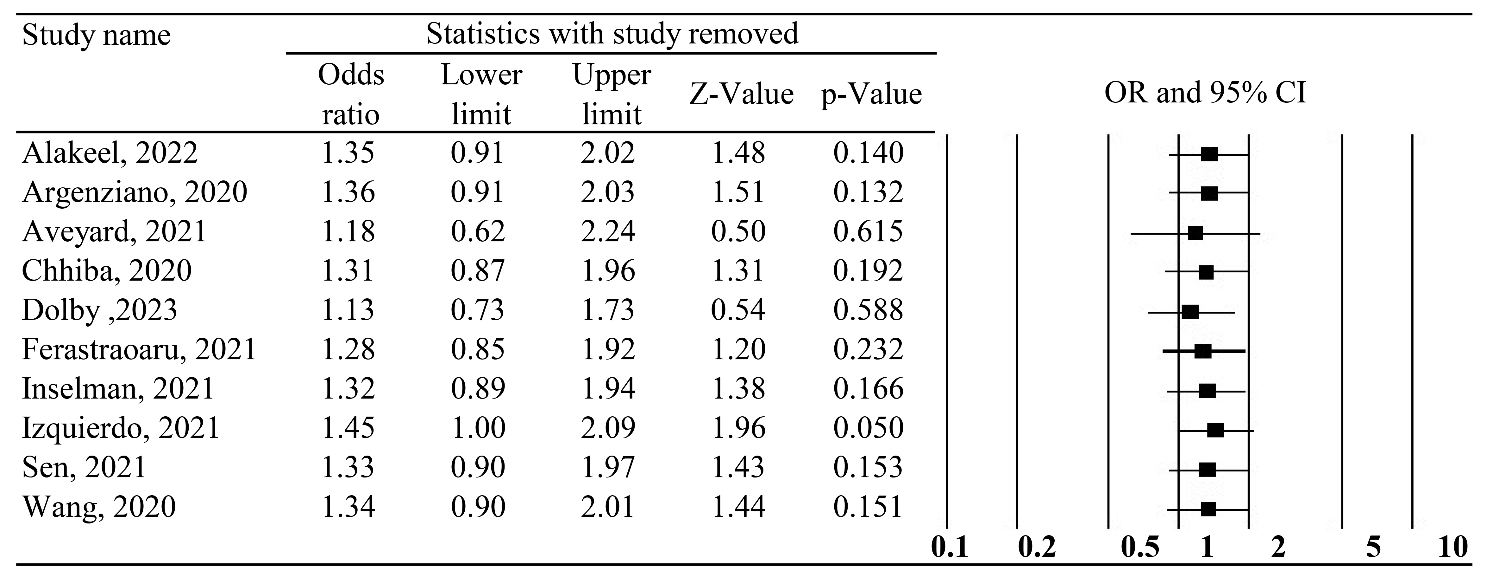


(D)

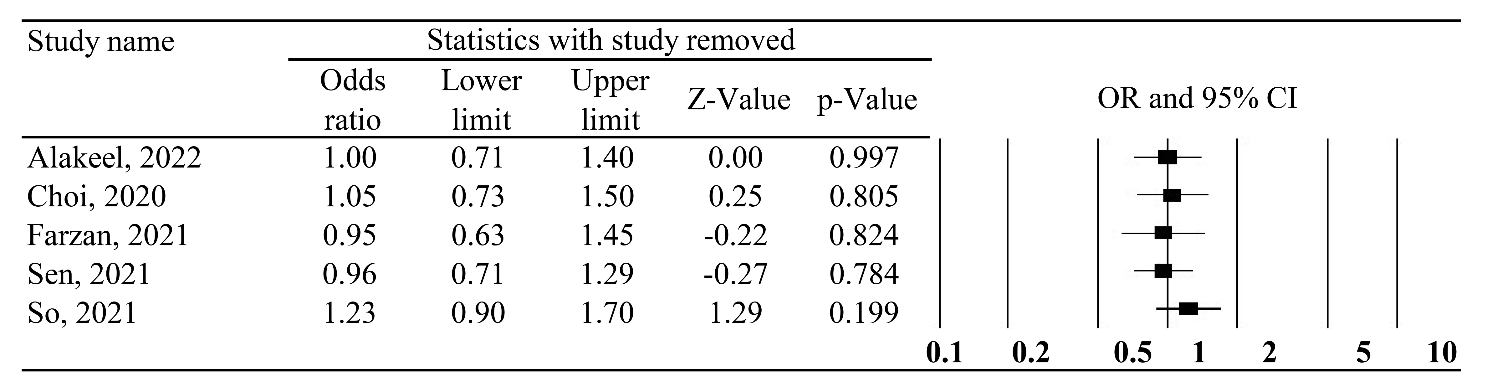


(E)


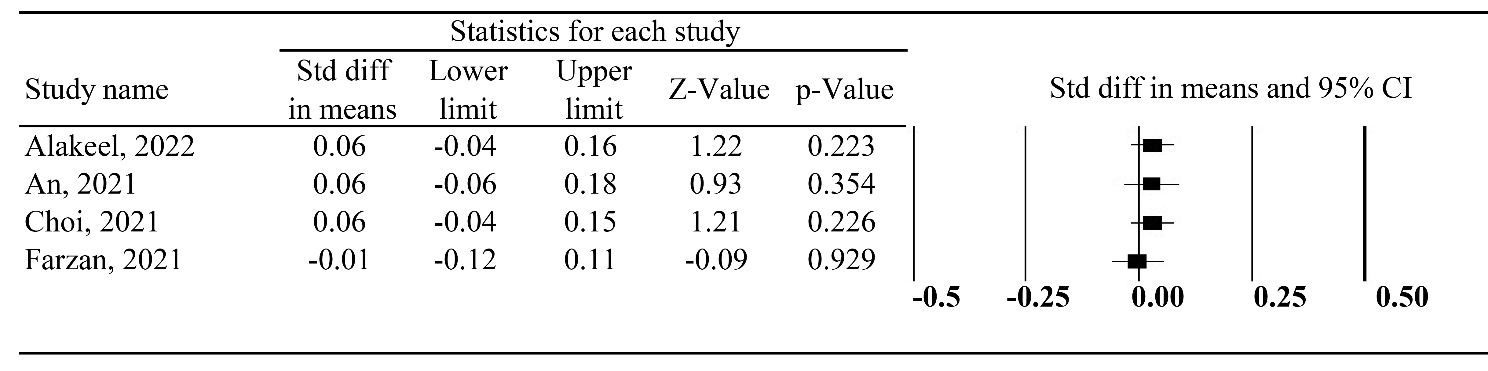

Supplement: S1 Fig — Leave-one-out sensitivity test of the risk of mortality (A), ICU admission (B), hospitalization (C), mechanical ventilation use (D) and length of hospital stay (E) between inhaled corticosteroid (ICS) use and non-use. (DOCX) [file pone.0295366.s001.docx]
