# Supplementary material for: The influence of prior use of inhaled corticosteroids on COVID-19 outcomes: A systematic review and meta-analysis
Source: PLoS One. 2024 Jan 19;19(1):e0295366. doi: 10.1371/journal.pone.0295366 (PMC10798539; doi:10.1371/journal.pone.0295366)
Supplement: S1 Table — (DOCX) [file pone.0295366.s002.docx]

**S1 Table. Search strategy**

Search strategy in **Pubmed** (Search date: 2023/02/28)

| **PICO** | **Search** | **Query** | **Items found** | **Time** |
| --- | --- | --- | --- | --- |
| **PI** | 26 | #10 AND (#20 OR #21 OR #25) | 4,355 | 11:16:02 |
|  | 25 | #22 OR #23 OR #24 | 104,041 | 11:15:58 |
|  | 24 | COPD | 104,041 | 11:15:51 |
|  | 23 | Chronic obstructive pulmonary disease | 95,707 | 11:15:44 |
|  | 22 | Pulmonary Disease, Chronic Obstructive [MeSH term] | 95,707 | 11:15:37 |
| **P** | 21 | Asthma [MeSH term] | 213,711 | 11:15:16 |
|  | 20 | #11 OR #19 | 18,560 | 11:15:04 |
|  | 19 | #12 AND (#13 OR #14 OR #15 OR #16 OR #17 OR #18) | 18,507 | 11:14:45 |
|  | 18 | Mometasone | 1,336 | 11:14:40 |
|  | 17 | Beclometasone | 4,049 | 11:13:51 |
|  | 16 | Fluticasone | 5,089 | 11:13:35 |
|  | 15 | Ciclesonide | 468 | 11:13:21 |
|  | 14 | Budesonide | 7,124 | 11:13:05 |
|  | 13 | corticosteroid* | 120,804 | 11:12:48 |
|  | 12 | inhal* | 181,472 | 11:12:38 |
| **I** | 11 | inhaled corticosteroid* | 14,835 | 11:12:30 |
|  | 10 | #1 OR #2 OR #3 OR #4 OR #9 | 363,029 | 11:12:21 |
|  | 9 | #5 AND (#6 OR #7 OR #8) | 5,689 | 11:12:14 |
|  | 8 | infection* | 2,362,145 | 11:12:10 |
|  | 7 | viral | 994,718 | 11:12:04 |
|  | 6 | virus | 1,398,839 | 11:11:56 |
|  | 5 | corona | 18,489 | 11:11:31 |
|  | 4 | sars-cov-2 | 193,705 | 11:11:25 |
|  | 3 | covid* | 331,197 | 11:11:20 |
|  | 2 | coronavir* | 157,120 | 11:11:12 |
| **P** | 1 | Coronavirus Infections [MeSH term] | 227,286 | 11:10:59 |

Search strategy in **Embase** (Search date: 2023/02/28)

| **PICO** | **No.** | **Query** | **Results** |
| --- | --- | --- | --- |
|  | #27 | #11 AND (#21 OR #22 OR #26) | 11114 |
|  | #26 | #23 OR #24 OR #25 | 196856 |
|  | #25 | copd | 109934 |
|  | #24 | 'chronic obstructive pulmonary disease' | 88004 |
|  | #23 | 'chronic obstructive lung disease'/exp [emtree term] | 169055 |
|  | #22 | 'asthma'/exp [emtree term] | 307788 |
|  | #21 | #12 OR #20 | 55699 |
|  | #20 | #13 AND (#14 OR #15 OR #16 OR #17 OR #18 OR #19) | 55699 |
|  | #19 | mometasone | 6387 |
|  | #18 | ciclesonide | 2042 |
|  | #17 | fluticasone | 21155 |
|  | #16 | budesonide | 27194 |
|  | #15 | beclometasone | 15048 |
|  | #14 | corticosteroid* | 389863 |
|  | #13 | inhal* | 278380 |
| **I** | #12 | 'inhaled corticosteroid*' | 19178 |
|  | #11 | #1 OR #2 OR #3 OR #4 OR #5 OR #10 | 427469 |
|  | #10 | #6 AND (#7 OR #8 OR #9) | 7333 |
|  | #9 | infection* | 3237687 |
|  | #8 | viral | 669466 |
|  | #7 | virus | 1766423 |
|  | #6 | corona | 25963 |
|  | #5 | 'sars-cov-2' | 136797 |
|  | #4 | covid* | 346895 |
|  | #3 | coronavir* | 359486 |
|  | #2 | 'coronavirus disease 2019'/exp [emtree term] | 295986 |
| **P** | #1 | 'coronavirinae'/exp [emtree term] | 117441 |

Search strategy in **Web of Science Core Collection** (Search date: 2023/02/28)

| **PICO** | **#** | **Query** | **Results** |
| --- | --- | --- | --- |
|  | #24 | #9 AND (#19 OR #20 OR #23) | 4698 |
|  | #23 | #21 OR #22 | 103034 |
|  | #22 | ALL=(COPD) | 77537 |
|  | #21 | ALL=('chronic obstructive pulmonary disease') | 68117 |
|  | #20 | ALL=(asthma) | 255238 |
|  | #19 | #10 OR #18 | 20504 |
|  | #18 | #11 AND (#12 OR #13 OR #14 OR #15 OR #16 OR #17) | 20504 |
|  | #17 | ALL=(mometasone) | 1725 |
|  | #16 | ALL=(ciclesonide) | 576 |
|  | #15 | ALL=(fluticasone) | 9082 |
|  | #14 | ALL=(budesonide) | 10699 |
|  | #13 | ALL=(beclometasone) | 336 |
|  | #12 | ALL=(corticosteroid*) | 119531 |
|  | #11 | ALL=(inhal*) | 140674 |
| **I** | #10 | ALL=(‘inhaled corticosteroid*’) | 16175 |
|  | #9 | #1 OR #2 OR #3 OR #8 | 428552 |
|  | #8 | #4 AND (#5 OR #6 OR #7) | 7224 |
|  | #7 | ALL=(infection*) | 1952793 |
|  | #6 | ALL=(viral) | 473751 |
|  | #5 | ALL=(virus) | 1065561 |
|  | #4 | ALL=(corona) | 63848 |
|  | #3 | ALL=('sars-cov-2') | 108935 |
|  | #2 | ALL=(covid*) | 381058 |
| **P** | #1 | ALL=(coronavir*) | 141528 |

Search strategy in **Cochrane Library** (Search date: 2023/02/28)

| **PICO** | **Search** | **Query** |
| --- | --- | --- |
| P | #1 | Coronavirus Infections [MeSH term] |
|  | #2 | coronavir* |
|  | #3 | covid* |
|  | #4 | sars-cov-2 |
|  | #5 | corona |
|  | #6 | virus |
|  | #7 | viral |
|  | #8 | infection* |
|  | #9 | #5 AND (#6 OR #7 OR #8) |
|  | #10 | #1 OR #2 OR #3 OR #4 OR #9 |
| I | #11 | inhaled corticosteroid* |
|  | #12 | inhal* |
|  | #13 | corticosteroid* |
|  | #14 | Beclometasone |
|  | #15 | Budesonide |
|  | #16 | Fluticasone |
|  | #17 | Ciclesonide |
|  | #18 | Mometasone |
|  | #19 | #12 AND (#13 OR #14 OR #15 OR #16 OR #17 OR #18) |
|  | #20 | #11 OR #19 |
|  | #21 | Asthma [MeSH term] |
|  | #22 | Pulmonary Disease, Chronic Obstructive [MeSH term] |
|  | #23 | Chronic obstructive pulmonary disease |
|  | #24 | COPD |
|  | #25 | #22 OR #23 OR #24 |
|  | #26 | #10 AND (#20 OR #21 OR #25) |

Cochrane Database of Systematic Reviews (CDSR): 144

Cochrane Central Register of Controlled Trials (CENTRAL): 398

Search strategy in **Scopus** (Search date: 2023/02/28)

(coronavir* OR covid* OR 'sars-cov-2' OR (corona AND (virus OR viral OR infection*))) AND ('inhaled corticosteroid*' OR (inhal* AND (corticosteroid* OR Beclometasone OR Budesonide OR Fluticasone OR Ciclesonide OR Mometasone)))

Result: 3,274 trials
